# A (PRES)sing Matter: Two Cases of Posterior Reversible Encephalopathy Syndrome (PRES) in Pregnancy and Postpartum

**DOI:** 10.7759/cureus.79255

**Published:** 2025-02-18

**Authors:** Jithin Reddy Vennapusa, Prabhav Kashyap Godavarthy, Shreya Garikipaty, Maitri Kulkarni, Sangeeta Chippa

**Affiliations:** 1 Department of Obstetrics and Gynaecology, Mamata Academy of Medical Sciences, Hyderabad, IND

**Keywords:** : eclampsia, emergency obstetric care, generalized tonic clonic seizures, hypertension in pregnancy, magnetic resonance (mr), obstetric acute medicine, posterior reversible encephalopathy syndrome (pres), postpartum eclampsia, pre-eclampsia

## Abstract

Posterior reversible encephalopathy syndrome (PRES) is a rare but serious neurological condition characterized by headaches, seizures, vision disturbances, and altered mental status. While it is commonly associated with hypertensive disorders, including preeclampsia and eclampsia, its exact pathophysiology remains unclear. We discuss the cases of two obstetric patients diagnosed with PRES: one in antepartum and the other during postpartum. Both exhibited severe hypertension and neurological symptoms, confirmed with neuroimaging, requiring urgent intervention with blood pressure control and seizure prophylaxis. Despite the reversible nature of PRES, a delay in diagnosis can lead to significant morbidity. These cases highlight the diverse clinical presentations of PRES and underscore the importance of early diagnosis and aggressive management to prevent complications. Through a discussion of potential underlying mechanisms, imaging findings, and treatment strategies, this report contributes to the growing understanding of PRES in pregnancy and postpartum, emphasizing the need for continued research and awareness.

## Introduction

Posterior reversible encephalopathy syndrome (PRES) is a rare clinical and radiological condition first described by Hinchey et al. in 1996 in the New England Journal of Medicine [1]. It is characterized by reversible subcortical vasogenic edema predominantly affecting the posterior, i.e., parieto-occipital, regions of the brain. Clinically, PRES presents with acute neurological deficits, including visual disturbances, seizures, headaches, and altered mental status. The condition is linked to various triggers such as hypertensive emergencies, eclampsia, immunosuppressive therapy, and systemic inflammatory states. Prompt detection of PRES is crucial, as early diagnosis and management of underlying causes typically leads to a complete reversal of symptoms and radiological findings [2]. While most patients experience recovery, PRES is not always fully reversible and can be associated with significant morbidity and even mortality [3]. Preeclampsia and eclampsia are likely among the most common causes of PRES, as many cases may remain undiagnosed and unreported due to the lack of imaging studies. Nonetheless, it is worth noting that it remains uncertain whether a true cause-and-effect relationship exists between PRES and preeclampsia/eclampsia, or if they are independent conditions with some degree of clinical overlap [4].

This case report aims to contribute to the growing body of literature on PRES by highlighting its occurrence in obstetric patients, particularly in the antepartum and postpartum periods. While PRES is well-documented in association with hypertensive disorders such as preeclampsia and eclampsia, its clinical presentations remain variable, leading to potential underdiagnosis. These cases emphasize the importance of recognizing PRES in diverse obstetric scenarios, as delayed diagnosis can result in significant morbidity. Furthermore, our report underscores the need for a standardized approach to early detection and management, particularly in low-resource settings where neuroimaging may not always be readily available. By examining two distinct presentations of PRES, we aim to provide additional insights into its pathophysiological mechanisms, diagnostic challenges, and therapeutic strategies, thereby filling gaps in the existing literature on its obstetric manifestations.

## Case presentation

Case 1

AS, a 26-year-old primipara presented with complaints of headache, which was throbbing and pounding in nature, and photophobia on the fifth postnatal day; however, there was no associated blurring of vision, nausea, or vomiting. The patient had a known history of hypothyroidism which was well-controlled on oral thyroxine. There was no history of any other comorbidities including seizures, or intake of oral contraceptives. Previously, she had been diagnosed with mild hypertension during her 30th week of gestation; she had been evaluated and started on oral antihypertensives (labetalol 100 mg, twice daily), regularly followed up, and her pressures had remained normal with treatment. On completion of 37 weeks of gestation, labor had been induced and she had delivered a 3000-gram female neonate vaginally with APGAR scores of 8 and 9 at one and five-minute intervals. She was tapered off the antihypertensives due to normal blood pressure readings, and discharged on the second postnatal day.

She had remained asymptomatic until the 5th postnatal day when she developed the symptoms described above and sought medical attention. Upon admission to the labor ward, the patient underwent a comprehensive examination. Her blood pressure was elevated at 160/100 mmHg, her heart rate was 90 beats per minute, and her oxygen saturation was 98%. Both obstetric and ophthalmic examinations were unremarkable.

Treatment commenced with a loading dose of 4 grams of intravenous (IV) magnesium sulfate, followed by 20 mg of IV labetalol. Half an hour later, the patient experienced a generalized tonic-clonic seizure (GTCS), prompting the administration of 2 milligrams of IV lorazepam. Following the seizure, the patient was in a postictal state, confused but arousable, with no signs of ongoing seizure activity. Cranial nerves were intact, with pupils equal and reactive, and no gaze preference. Motor examination showed no focal weakness, normal tone, and symmetric reflexes. The sensory assessment was limited due to confusion but appeared grossly intact. Coordination could not be reliably assessed. No meningeal signs were present. The postictal state was observed to be resolving.

The treatment with IV magnesium sulfate was continued according to the Zuspan regimen; a total of 100 mg IV labetalol was administered in divided doses. Another convulsive seizure occurred after 60 minutes, which prompted the administration of IV sodium valproate (500 mg BD) and IV levetiracetam (500 mg). The patient was monitored for magnesium toxicity and the therapeutic target of blood pressure was <150/100 mmHg.

**Figure 1:**
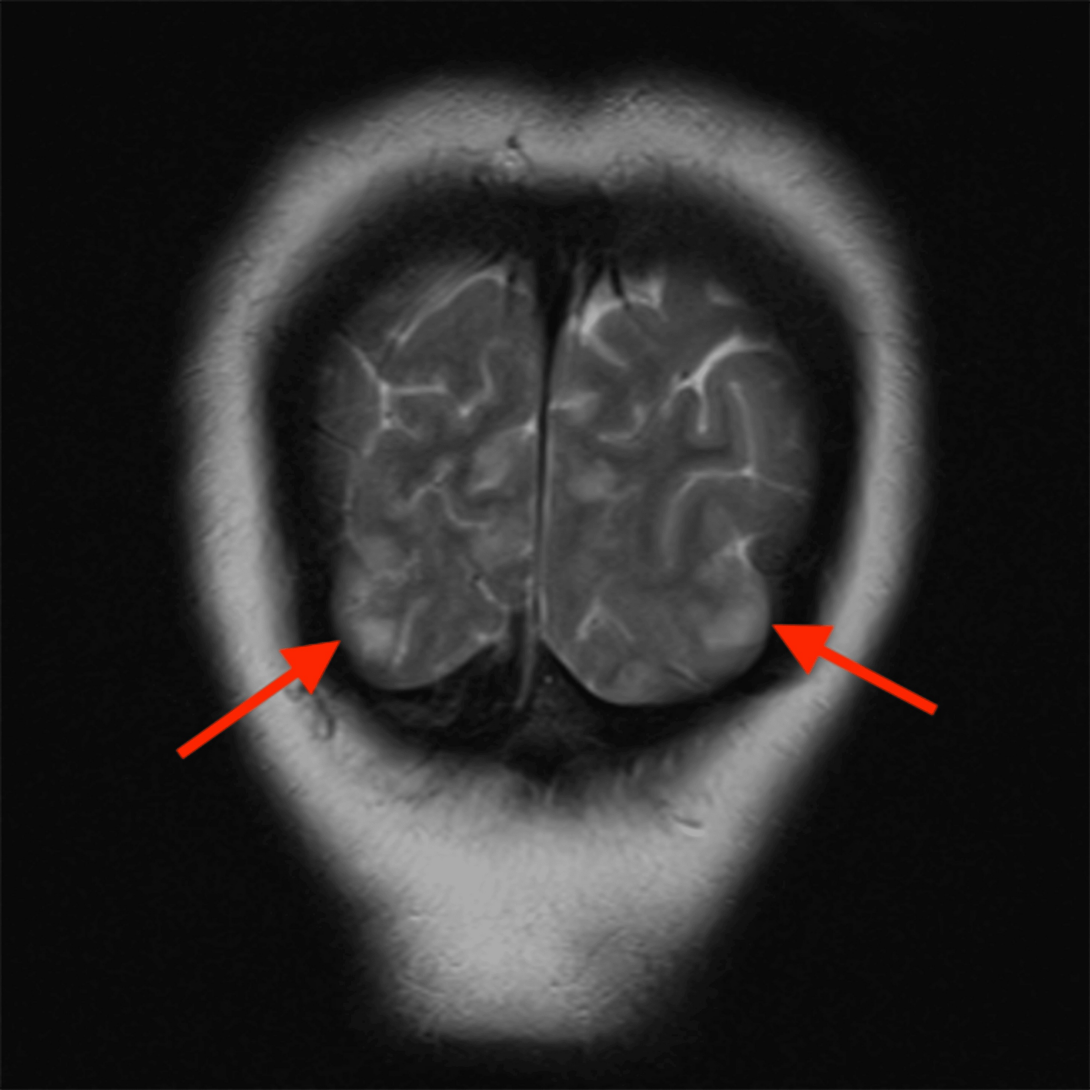
Coronal T2 MRI of Patient 1 (AS) demonstrating hyperintensities (arrows) in bilateral parietal regions The red arrows point to bilateral parietal regions with patchy, symmetrical cortical and subcortical T2 hyperintensities, consistent with PRES MRI: magnetic resonance imaging; PRES: posterior reversible encephalopathy syndrome

**Figure 2:**
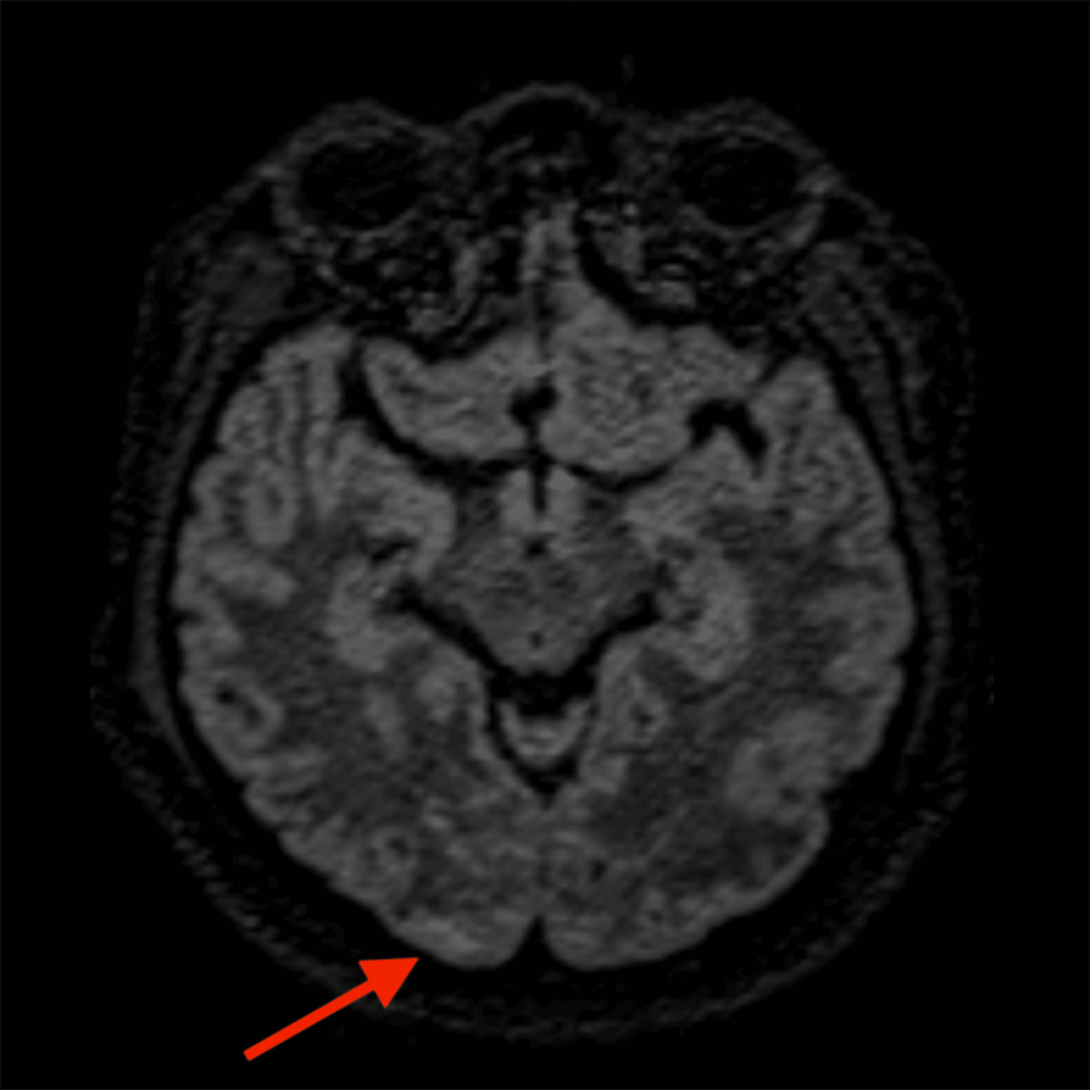
Axial FLAIR image of Patient 1 (AS) demonstrating hyperintensity (arrow) in the occipital region The red arrow points to the occipital region with patchy FLAIR hyperintensity, consistent with PRES FLAIR: fluid-attenuated inversion recovery; PRES: posterior reversible encephalopathy syndrome

Laboratory studies were conducted to elucidate the underlying cause of the patient’s symptoms. These studies revealed positive urinary proteins and ketones, hemoglobin of 9.5 mg/dl, potassium of 3.4 mmol/l, alkaline phosphatase (ALP) of 122 U/L, and uric acid of 9.2 mg/dl. The remaining laboratory tests were unremarkable. Given multiple convulsions, to rule out other neurological conditions such as hemorrhage and space-occupying lesions, an MRI of the brain with venography was performed. The imaging revealed patchy, symmetrical cortical and subcortical T2/FLAIR hyperintensities in bilateral posterior parietal and occipital lobes, consistent with PRES. The final diagnosis was postpartum eclampsia leading to PRES (Figures 1, 2).

Based on the diagnostic findings, the patient was transferred to the ICU and managed according to the established treatment regimen, which included IV labetalol, magnesium sulfate, and seizure prophylaxis with levetiracetam. She was discharged from the hospital on the fifth day of admission, on oral nifedipine (40 mg once daily), levetiracetam (500 mg twice daily), and a combination of metoprolol (50 mg) and amlodipine (5 mg) once daily. She was counseled about warning signs (headache, vision changes, seizure), blood pressure monitoring, and medication compliance. The patient was lost to follow-up.

Case 2

MN, a 21-year-old primigravida, of 24 weeks and five days gestation, with a known history of gestational hypertension, presented to the emergency labor ward after experiencing one episode of GTCS at home one hour prior. She had complained of a headache, nausea, and vomiting preceding the seizure. She did not complain of any visual changes. There was also no history of any other comorbidities including seizures. Upon arrival, the patient was in a postictal state, confused but arousable, with no signs of ongoing seizure activity. Cranial nerve examination was intact, with pupils equal and reactive and no gaze preference. Motor examination revealed no focal weakness, normal tone, and symmetric reflexes. The sensory assessment was limited due to confusion but appeared grossly intact. Coordination could not be reliably assessed. No meningeal signs were present. The postictal state was observed to be resolving. Her blood pressure was elevated to 155/110 mmHg, and she was tachycardic at 120 beats per minute.

The patient was receiving oral labetalol 100 mg twice daily for her hypertension but had been non-compliant with the medication for two weeks before presentation and was irregular with her antenatal checkups since conception. She had not received any other oral medication. A loading dose of 4 grams of IV magnesium sulfate was administered according to the Zuspan regimen, along with 20 mg of IV labetalol in divided doses, and 500 mg of IV levetiracetam was initiated. The patient was monitored for magnesium toxicity and the therapeutic target of blood pressure was <150/100 mmHg. Laboratory studies revealed positive urine ketones and protein, hemoglobin of 7.8 mg/dL, white blood cell count of 14,000 cells/mm^3^, total serum protein of 5.8 g/dL, serum uric acid of 10.5 mg/dL, and lactate dehydrogenase (LDH) of 290 U/L. Renal and liver function tests were otherwise unremarkable.

**Figure 3:**
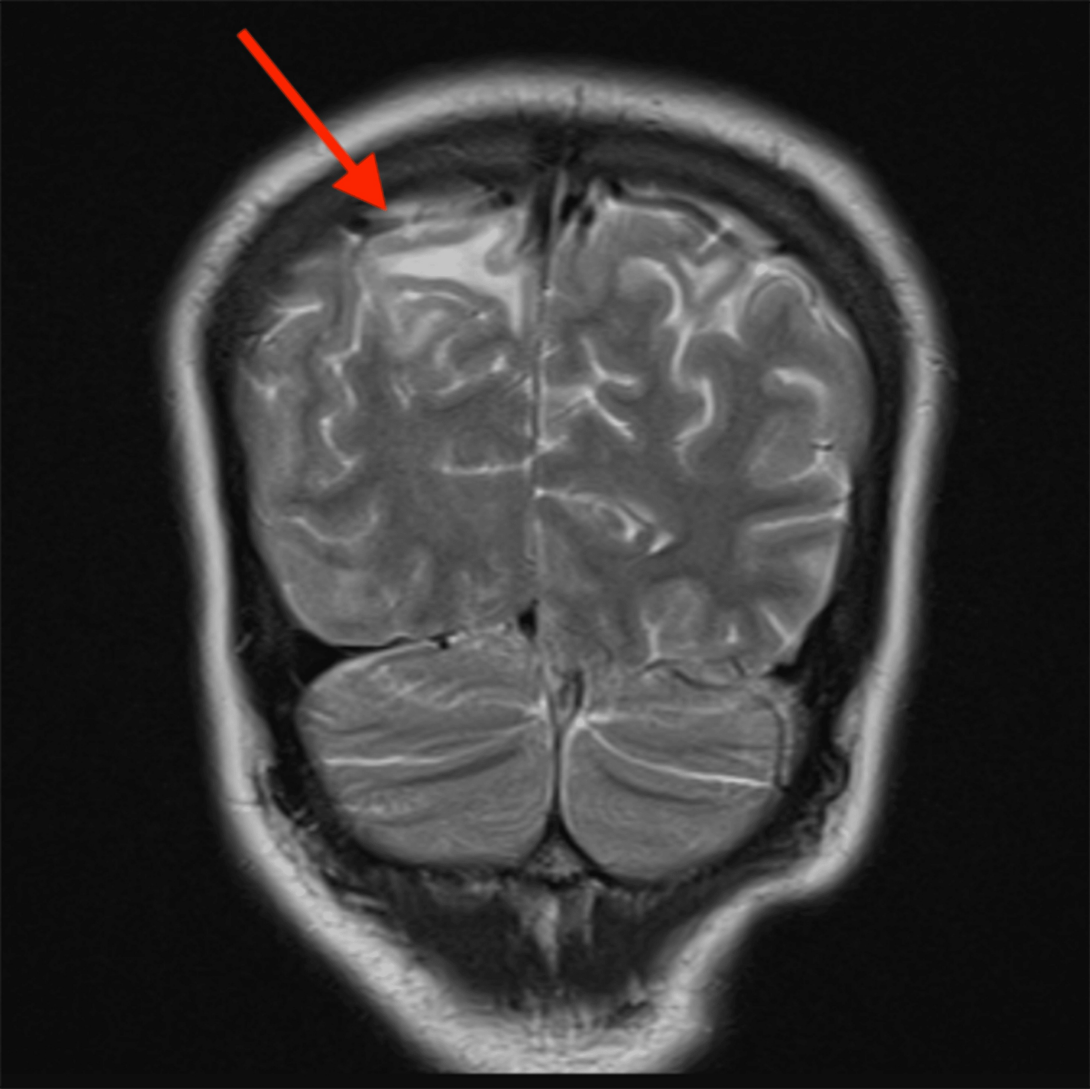
Coronal T2 MRI of Patient 2 (MN) showing hyperintensity (arrow) in the parietal region The red arrow points to the parietal region with patchy, symmetrical cortical and subcortical T2 hyperintensities, consistent with PRES MRI: magnetic resonance imaging; PRES: posterior reversible encephalopathy syndrome

**Figure 4:**
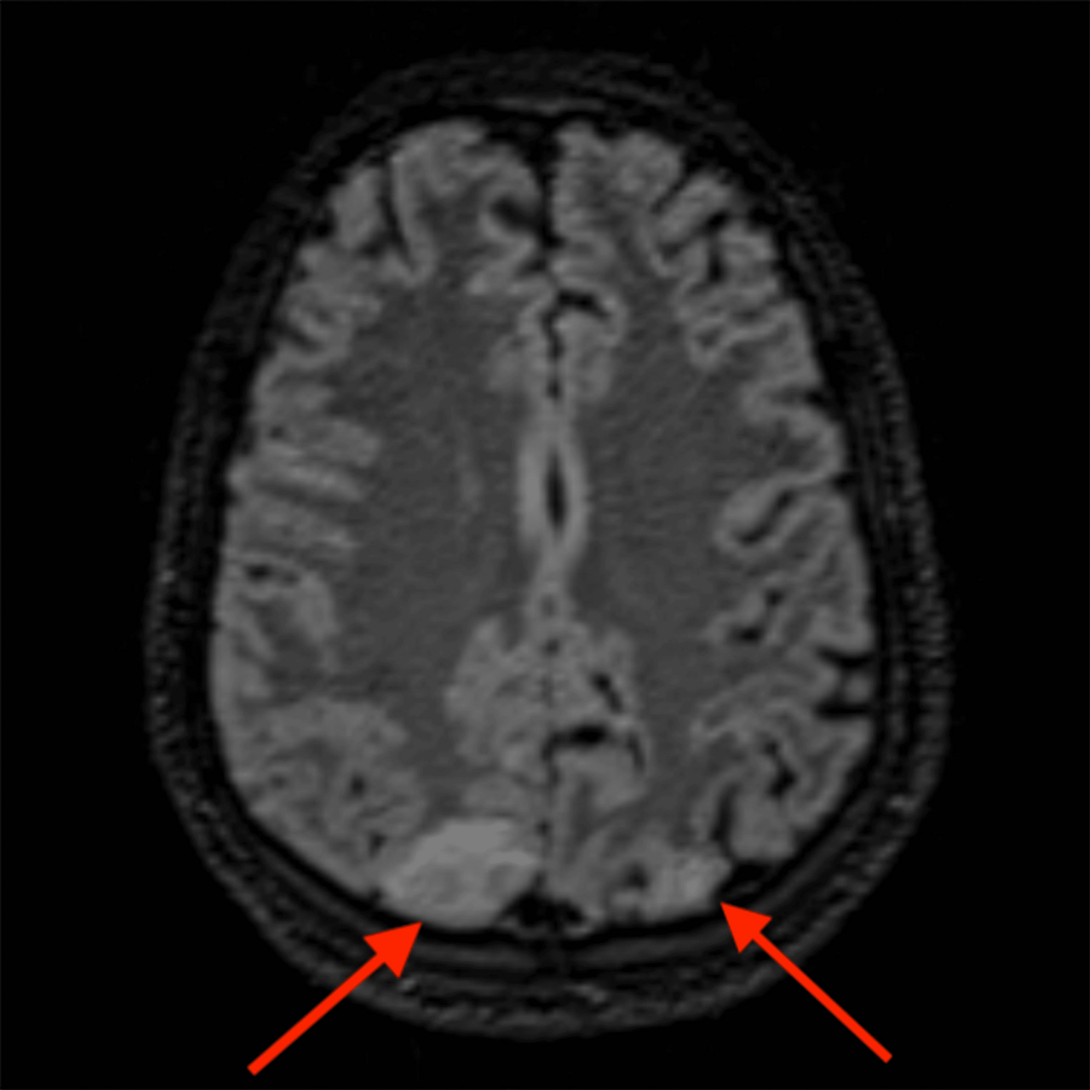
Axial FLAIR image of Patient 2 (MN) showing hyperintensities (arrows) in bilateral posterior parietal regions The red arrows point to bilateral posterior parietal regions with patchy FLAIR hyperintensities, consistent with PRES FLAIR: fluid-attenuated inversion recovery; PRES: posterior reversible encephalopathy syndrome

Given the patient's incoherence and unresponsiveness to verbal commands following treatment, and to exclude the possibility of other neurological conditions such as hemorrhage and space-occupying lesions, a brain MRI with venogram was ordered. The imaging revealed patchy, symmetrical cortical and subcortical T2/FLAIR hyperintensities in bilateral posterior parietal lobes. These findings, in conjunction with the clinical and laboratory findings of high blood pressure and seizures with proteinuria, were consistent with eclampsia leading to PRES (Figures 3, 4).

Preterm induction of labor and delivery were decided upon considering worsening maternal status and fetal compromise. An 875-gram male neonate was delivered with one and five-minute APGAR scores of 1 and 4, respectively. The neonate was admitted to the NICU for respiratory and hemodynamic support, and the prognosis was guarded due to extreme prematurity.

The patient was managed in the ICU and made a full recovery and was subsequently discharged seven days after admission. She was prescribed oral levetiracetam (500 mg twice daily) and a combination of metoprolol (50 mg), telmisartan (40 mg), and cilnidipine (10 mg) once daily for blood pressure control. The patient was counseled regarding blood pressure monitoring and medication compliance. The patient was lost to follow-up.

## Discussion

PRES is a clinicoradiological condition characterized by diverse neurological symptoms that may include headache, visual impairment, altered mentation, drowsiness, seizures, and characteristic neuroradiological findings [5]. The cases presented here highlight the diverse presenting symptoms and the variable timing of the onset of PRES. Numerous clinical conditions like preeclampsia, eclampsia, acute or chronic renal diseases, endocrine dysfunctions like primary aldosteronism, electrolyte imbalances like hypercalcemia, and the use of cytotoxic drugs have been linked to the development of PRES [6]. Preeclampsia and eclampsia are closely associated with the development of PRES. One study revealed that 97.9% of eclamptic patients had PRES on neuroimaging studies, indicating that PRES might be a core element in the pathogenesis of eclampsia [7].

Our two cases add to the ongoing discourse on hypertension and headaches in PRES. Both had histories of gestational hypertension. Patient 1 (AS) developed a throbbing, pounding headache accompanied by photophobia five days into the postpartum period after an uncomplicated delivery. Meanwhile, Patient 2 (MN) presented during the antepartum period following an episode of generalized tonic-clonic seizures, preceded by classic warning signs - headache, nausea, and vomiting. Upon presentation, both patients had elevated blood pressures - 160/100 mmHg and 155/110 mmHg, respectively.

The exact mechanisms underlying this condition remain unclear. Elevated blood pressure and endothelial injury seem to be almost always present. Vasoconstriction, which is a compensatory response to hypertension, causes vasogenic and cytotoxic edema and is thought to play a key role in the development of clinical symptoms and neuroradiological findings [8]. Another theory suggests that the development of PRES might involve the activation of the immune system leading to the release of certain cytokines like tumor necrosis factor (TNF) and interleukin-1 (IL-1), which trigger endothelial damage and fluid leakage [9]. Another recently published theory suggests that conditions like eclampsia are related to arginine vasopressin hypersecretion, which in turn can lead to cerebral vasoconstriction, endothelial dysfunction, and ischemia, leading to cytotoxic edema [10]. A systematic review and meta-analysis identified a link between subclinical hypothyroidism and increased risk of preeclampsia [11]. Patient 1 (AS) had a history of hypothyroidism and it was effectively managed with oral thyroxine therapy. 

Regarding diagnostic modalities, MRI is the gold standard. The most common finding on MRI in patients of PRES is the presence of subcortical and cortical edema, predominantly affecting the parieto-occipital regions. In Patient 1 (AS), MRI revealed symmetrical cortical and subcortical T2/FLAIR hyperintensities in the bilateral posterior parietal and occipital lobes, consistent with the classic finding in PRES. Similarly, Patient 2’s (MN) MRI showed patchy T2/FLAIR hyperintensities in the bilateral posterior parietal and occipital lobes, involving both cortical and subcortical white matter, further supporting the diagnosis. While classic imaging findings were seen in both of our patients, a few studies have shown that PRES may show atypical involvement limited to the deep gray nuclei like the thalamus and basal ganglia, brain stem, cerebellar hemispheres, or rarely, the spinal cord, without affecting the cerebral hemispheres [12-14]. Moreover, there may be underreporting of PRES due to resource constraints in low- to middle-income countries. 

Currently, there are no clinical trials or standardized treatment regimens available exclusively focusing on PRES. The mainstay of management revolves around control and treatment of the contributing conditions. Around 40% of patients diagnosed with PRES require intensive care and management as the chances of complications like status epilepticus, cerebral ischemia, intracranial hemorrhage or elevated intracranial tension are quite high [15].

## Conclusions

This case report highlights the critical need for early recognition and timely intervention in managing PRES in obstetric patients to prevent severe complications and ensure optimal recovery. It underscores the complex relationship between hypertensive disorders, neurological manifestations, and maternal-fetal risks, emphasizing the necessity of close monitoring, particularly in patients with pregnancy-induced hypertension or endocrine abnormalities. These cases demonstrate the diverse clinical presentations and pathophysiological mechanisms of PRES, reinforcing the importance of an interdisciplinary approach to diagnosis and management. Establishing standardized protocols for early detection and management in obstetric settings is crucial. Additionally, these cases contribute to the growing body of literature on PRES, highlighting the need for further research to refine therapeutic strategies and improve patient outcomes in this potentially life-threatening condition. A limitation of our study is the loss of both patients to follow-up, preventing assessment of long-term neurological complications of PRES in pregnant patients, which may differ from those in non-pregnant patients.
